# What motivates British parents to consent for research? A questionnaire study

**DOI:** 10.1186/1471-2431-7-12

**Published:** 2007-03-09

**Authors:** Helen M Sammons, Maria Atkinson, Imti Choonara, Terence Stephenson

**Affiliations:** 1 Academic Division of Child Health, University of Nottingham, The Medical School, Derbyshire Children's Hospital, Uttoxeter Road Derby, DE22 3DT, UK; 2 Department of Child Health, University of Nottingham, B Floor, Queens Medical Centre, Nottingham, NG7 2UH, UK

## Abstract

**Background:**

Informed consent is the backbone of a clinical trial. In children this is given by their parents. There have been many studies in the neonatal population but little is known about the views of the parents of infants and young children from within the United Kingdom. The objectives of this study were to assess what motivates parents to consent to a randomised clinical trial (RCT), their feelings on consent and participation and the factors that would influence their decision to take part in a future study.

**Methods:**

The setting was a multi-centre randomised but non-blinded equivalence trial of oral versus intravenous (IV) treatment for community acquired pneumonia in previously well children aged 6 months to 16 years in the UK (PIVOT Study). Parents were sent a postal questionnaire at the end of the study which included open and closed-ended questions. Fishers Exact Test was used to analyse associations in non parametric categorical data.

**Results:**

243 children were recruited into the PIVOT study. Of a possible 235, 136 questionnaires were returned (response rate 59%). Of those questionnaires returned; 98% of parents remembered consenting, 95% felt they were given enough time to make their decision and 96% felt they received enough information. Major reasons for participation were benefit to other children in the future 31%, contribution to science 27%, benefit to their own child 18%. Most parents (85%) did not feel obliged to participate. 62% felt there was an advantage to taking part and 18% felt there was a disadvantage. 91% of parents said they would take part in a similar study in the future, stating influences on their decision being benefit to their own child (91%) and benefit to all children (89%).

**Conclusion:**

The major motivation in parents consenting for their previously well child to participate in an RCT of therapy for an acute medical illness was to increase medical knowledge in the future. Most saw an advantage in taking part in the trial and did not feel obliged to participate.

## Background

Research in a child is different to that in adults. Informed consent is essential for recruitment into a randomised controlled trial. The informed consent process is undertaken in the majority of paediatric trials by the child's parent. It has been found that a child's ability to assent or consent to research under the age of 9 years is limited [1]. Recruitment is said to be difficult within paediatric trials and quoted as being the single most difficult problem to overcome; leading to delays, increased costs and failure to complete drug trials [2-4]. Therefore understanding why a parent allows their child to participate in research is essential in taking forward paediatric research in the future.

There have been several studies on the consent process and the information retained by parents following this process. The majority of work within the United Kingdom has centred on neonatal research[5,6]. A study in the Netherlands[7] of a randomised placebo controlled trial of ibuprofen syrup to prevent febrile convulsions found that, within the infant age group, the major factors in parents granting approval were contribution to clinical science (51%) and benefit to the child (32%). A quarter of parents felt obliged to participate and over half (60%) said they would be willing to participate in a similar study in the future.

Based on the information provided by the clinician, parents decide whether or not to permit their child to participate in that study. Understanding the parent's major thoughts and motivations at this time may help improve this process and increase the numbers participating in future studies. This present study aims to look at a group of parents approached for consent in a multi-centre randomised controlled trial of previously well infants and children presenting with pneumonia, within the United Kingdom[8]. The aim was to see if the motives of British parents are similar to those seen in Europe, to assess parental views on the informed consent process, the information provided and their reasons for taking part in the study. Their willingness to participate in future research was also examined.

## Methods

### The Trial

A multicentre randomised controlled equivalence trial compared oral amoxicillin and IV benzylpenicillin for community acquired pneumonia in children in hospital (PIVOT)[8]. It was undertaken at eight hospitals in England. Children aged 6 months to 16 years with fever, respiratory symptoms or signs and radiologically confirmed pneumonia were eligible. 245 children were randomised to either oral amoxicillin or IV benzylpenicillin. The primary outcome measure was time for the temperature to be less than 38°C for 24 continuous hours and oxygen requirement to cease.

### Informed consent procedure

Once the diagnosis had been made the parents were approached regarding the trial. Written parent information was provided at the child's diagnosis and the parent was then asked to reach a decision on participation prior to the start of treatment. Information sheets were provided for children aged 7 years and over. Older children and teenagers were asked for their assent and could complete the consent form as well as their parents. Unlike studies of children's cancer, for example, when parents may have 24 hrs to reflect, consent had to be decided rapidly as treatment could not be deferred. All children had blood tests performed and those in the intravenous group had a cannula left in situ for antibiotics. If the parents declined consent and volunteered a reason, this was recorded on a separate data collection form anonymously.

### The Questionnaire

**Figure 1:**
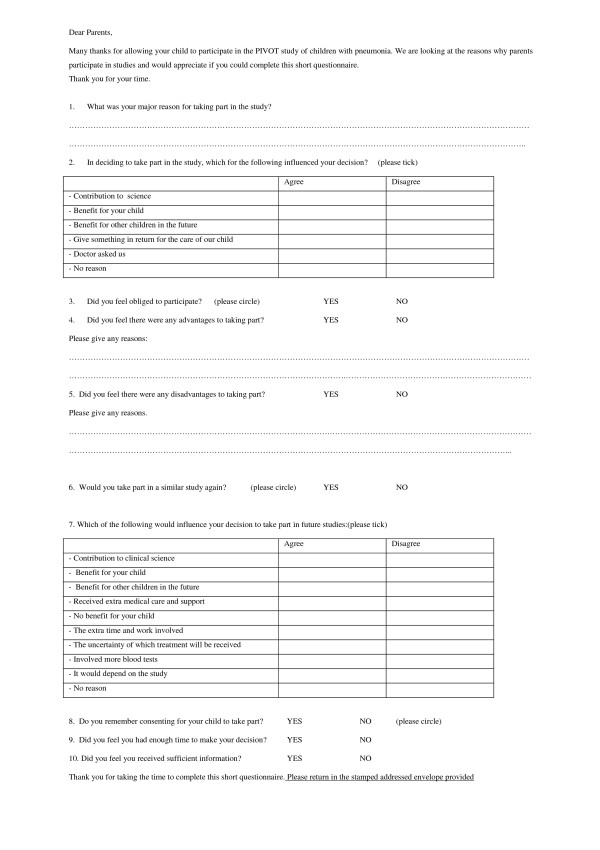
Original Questionnaire.

Ethical approval was obtained from the Southern Derbyshire Local Research Ethics Committee. A parental questionnaire was designed to try and elicit parental views on consent and participation in research. The questionnaire included both structured and semi-structured questions as detailed below (Figure 1). This was kept to two sides of A4 paper to try and encourage return. Because of this the questionnaire was limited to include the primary question on reason for participation. Demographic details on the person completing the questionnaire were not collected.

The themes for reasons for participation were taken from a previous questionnaire based study conducted with parents in the Netherlands[7] as these had already been validated in a European population. Parents were asked to agree or disagree with a list of reasons that might have influenced their decision to enrol their child in the study, such as benefit to all children in the future (Figure 1- Questionnaire). Open free text questions were posed for the main reason for participation and any advantages or disadvantages they experienced from the study. The questionnaire was not piloted as there were no parents available in hospital who had taken part in research studies at the time of its development.

The anonymous questionnaire was mailed to all parents in July 2004 after the two-year recruitment period had finished. A stamped addressed envelope was included. It was felt not to be appropriate to send questionnaires to the two parents who withdrew during the study. The questionnaire was re-mailed in September 2004. A returned completed questionnaire was taken as consent by the parent. Completed questionnaires were coded and analysed using the SPSS statistical package. Fishers Exact Test was used to analyse association in non parametric categorical data.

## Results

### Demographics

243 parents were identified from the PIVOT study after the two year recruitment had finished. Eight addresses were incorrect and therefore questionnaires were unable to be delivered. 136 questionnaires were returned (30 after the re mailing), a response rate of 59%. The response rate was not affected by time, with a constant response rate across the whole duration of the study. The median age of the child for which parents responded to the questionnaire was 2.0 years (range 6 months to 12 years and 4 months) compared to a median age of 2.4 years in the overall study. The questionnaire did not specify which parent was filling in the questionnaire. The PIVOT study had a 69% consent rate and details on the socioeconomic status of the families within the study or the questionnaire were not collected.

### Reasons for participation

**Figure 2:**
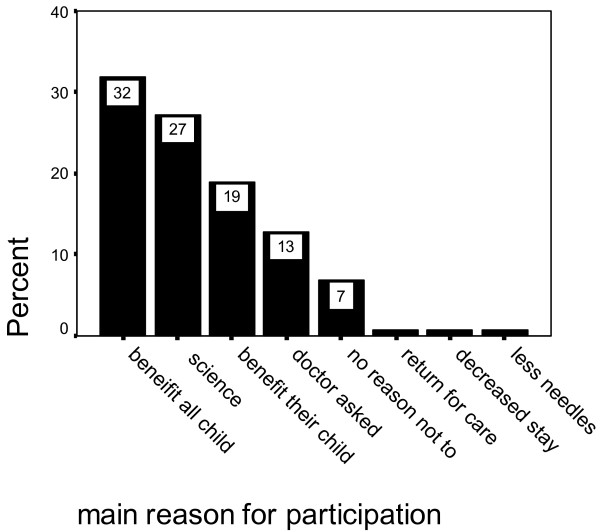
Main reason given by parents for their participation.

The major reason given by parents for taking part in the study was benefit to all children in the future and a contribution to science in 57% (Figure 2). Only 18% said that the major reason they took part was benefit to their own child. Parents answered this question in their own words. The remainder stated that they participated because they were asked by a doctor or that there was no reason not to. When questioned directly on each theme, 96% agreed that benefit to all children and 72% benefit to their own child had influenced their decision to participate. 67% said that they consented in order to give something in return for care for their child and 63% because the doctors asking had influenced their decision. This was not statistically influenced by the child's age.

### Advantages and disadvantages of participation

Just under two thirds (62%) felt that there had been an advantage to taking part in the study. Of those that expressed an advantage, for over half (57.5%) this was theoretical in giving knowledge for treatment of all children in the future. The remainder felt the benefit was to their own child including oral medication, quicker recovery, more information given and closer monitoring.

24 parents (18%) felt that there had been a disadvantage in participating. The disadvantages stated were the use of needles (although it had been explained that both groups would have blood tests as standard), delay in starting treatment due to randomisation, anxiety that randomised treatment would be less effective and having no choice over treatment. Four parents felt their child had a poorer recovery due to oral treatment (though this was not born out in the overall results which showed equivalence of the two treatments[8]). 18 parents (13%) said that they felt obliged to take part and 9 (7%) felt that they would not take part in a similar study in the future. 98% of parents remembered consenting, 95% felt that they had enough time to make their decision. 96% felt that they had received enough information. These answers were not statistically influenced by the child's age or the time since recruitment had taken place.

### Future studies

Parents were asked which factors would influence their decision to take part in a future study with their child. The major factors were benefit to their own child (91%), benefit to all children (89%) and contribution to medical science (83%). Interestingly only 14% said they would not take part if there was no benefit to their own child and 23% if it involved blood tests. The uncertainty of treatment would influence just under a third of parents' decisions but the majority (80%) said that it would depend on the design of the trial.

If a parent expressed an advantage, 62% (p = 0.002), or did not express a disadvantage, 81% (p = 0.05), then they were statistically more likely to say that they would take part in a similar study in the future. However 42 (84%) of those parents who did not feel there was an advantage would still take part in a similar future study. This was not statistically influenced by the child's age.

The relationship between wanting to take part in a similar study in the future was examined in relation to the views expressed on motivations for future studies. Parents who did not feel that 'benefit for other children in the future' influenced their decision to participate were less likely to participate again in the future (p = 0.045). If a parent felt that they did not receive enough information (p = 0.053), or have enough time to make their decisions (p = 0.004) then they were statistically more likely to express a wish not to participate in future studies. Randomisation to oral or IV treatment did not make a difference to this decision (p = 0.28).

### Declined to participate

43 parents approached to participate in the PIVOT trial declined for their child to take part. Of these 30 expressed a reason to the clinician and these were collected anonymously. The majority (25) stated that they wanted a specific treatment for their child, either IV (20) or oral (5). Many parents expressed a view that IV treatment was superior and therefore were unwilling to undergo randomisation. Of the remaining parents, two declined consent because they did not want to participate in a trial, two expressed that they were too distressed by their child's admission to consider consenting and one declined consent because of the paragraph on the consent form saying that the ethics committee would have access to their child's notes.

## Discussion

In this study, of a general paediatric condition in the childhood population in the UK, the major motivator for the participation in clinical trials is for the good of all children and the furthering of clinical science. This is a positive finding and good for future trial recruitment. It means that if a trial is designed well with a clear clinical question with which parents can identify, then they are likely to consider taking part. As an important predictor of consent this had previously been recognised in work carried out on clinical anaesthesia and surgical studies in children[9]. This is reinforced in our results in that parents who felt that they did not receive enough time to consider their decision or receive enough information were statistically less likely to wish to take part in future studies. The main reason expressed by those parents who declined to consent was the perception that one treatment was superior to another. This may show a lack of understanding of the information presented and this has been found to be another important predictor of consent[9]. The PIVOT study recruitment rate was 82% which was high compared to that quoted in previous studies of 68% and 43%[10]. This may be related to the pragmatic nature of the trial and the fact that it did not ask for any extra blood taking or immunisation, which have been quoted as reasons for non-enrolment in other studies[10].

The attitudes of our British parents were very similar to those seen in the previous Dutch study[7] with contribution to clinical science being the biggest motivator in both groups. A more recent study[11] in France showed that the possibility of receiving the most advanced treatments and the confidence placed in the medical team were their highest motivations for participation. Their population were children treated for either a cancer or HIV infection. The relatively high risk nature of these diseases is most likely the reason for the difference between their results and those seen in our own population. In a study conducted in the United States[12] the importance of receiving the newest drugs, financial benefit, and free office visits were highlighted. A statistical correlation was noted between the importance of free medication and lower family income. Financial incentives can be offered within the USA; in the study above the mean compensation was $570 per child recruited. This reflects the differences in the health care systems between the USA and the UK, where there is free access to health care. In our study, over two thirds of our parents responded that giving something in return for their care had influenced their decision to participate.

One of the limitations of the present study, and the questionnaire method, is the response rate (59% in our current study). This was lower than the previous Dutch study of 79%[7]. Of those who did not respond we do not know if their views would be similar to those who were willing to fill in the questionnaire. This may mean that we overestimate the positive attitudes to this and future studies. There would also have been a recall bias associated with mailing the questionnaire to all parents at the end of the study. Some parents may have been recruited to the trial up to two years prior to the questionnaire being completed and therefore their recall would have been different to those recruited in the last few months. There was however an equal spread of responses over the recruitment period and the time since recruitment did not statistically alter the responses to the questions of information, time and remembering consent. Our results are based on the responses to a single study, in a general paediatric condition, and thus the results may not be generalizable to other studies that have different risk/benefit profiles.

## Conclusion

This study has highlighted that the reasons that parents consent for clinical trials in the United Kingdom is similar to that seen in other European countries. Future work, ideally within a multinational trial of the same disease profile, to compare parental attitudes within different health care systems would be interesting. The majority of parents consent because they see the clinical need of the trials to answer questions for the treatment of future children. When financial incentives and cost of health care are introduced this may change motives. It is important that we understand the motivation of parents, within our own populations and different disease profiles, with the introduction of European legislation to ensure that all medicines are studied in children and the challenges that it will bring.

## Competing interests

The author(s) declare that they have no competing interests.

## Authors' contributions

HMS conceived the questionnaire study and designed, distributed, analysed the questionnaire and drafted the manuscript. MA was the research fellow for the PIVOT study, participated in the design of the questionnaire and gave comments on the manuscript. IC helped conceive the idea for the questionnaire and draft the manuscript. TS conceived the idea for PIVOT study, its design and analysis, commented on questionnaire design and the manuscript. All authors read and approved the final manuscript.

## Pre-publication history

The pre-publication history for this paper can be accessed here:
